# Anti-Müllerian hormone levels in patients with gestational trophoblastic neoplasia treated with different chemotherapy regimens: a prospective cohort study

**DOI:** 10.18632/oncotarget.23027

**Published:** 2017-12-06

**Authors:** Xiaoning Bi, Jingjing Zhang, Dongyan Cao, Hengzi Sun, Fengzhi Feng, Xirun Wan, Yang Xiang, Ling Qiu, Xinqi Cheng, Jiaxin Yang, Keng Shen

**Affiliations:** ^1^ Department of Obstetrics and Gynecology, Peking Union Medical College Hospital, Chinese Academy of Medical Sciences and Peking Union Medical College, Beijing, 100730, China; ^2^ Department of Clinical Laboratory, Peking Union Medical College Hospital, Chinese Academy of Medical Sciences and Peking Union Medical College, Beijing, 100730, China

**Keywords:** anti-Müllerian hormone, gestational trophoblastic neoplasia, follicle-stimulating hormone, chemotherapy

## Abstract

**Purpose:**

To assess the ovarian reserve of patients with gestational trophoblastic neoplasia (GTN) treated with chemotherapy by evaluating serum anti-Müllerian hormone (AMH) and follicle-stimulating hormone (FSH) levels before, during, and after chemotherapy.

**Results:**

The basal AMH level (mean: 3.98 ± 3.20 ng/mL) negatively correlated with age, while the basal FSH level (mean: 5.71 ± 9.69 mIU/mL) had no correlation with age. After 3 chemotherapy cycles, serum AMH levels decreased and FSH levels increased. The magnitude of the AMH level decline was significantly greater for combination chemotherapy than for single-agent dactinomycin D therapy (61.80% vs. 27.57%) (*p* = 0.0004) and was higher in patients whose regimens included etoposide (73.69% vs 40.51%) (*p* = 0.0359). After chemotherapy completion, AMH levels showed a further decline, and cumulative AMH concentration change was associated with doses of vincristine (*p* = 0.009) and etoposide (*p* = 0.032). At the 3-month follow-up, AMH levels significantly increased in the dactinomycin D group (*p* = 0.0067).

**Materials and Methods:**

This prospective study included 34 patients with GTN. Serum AMH and FSH levels were measured before chemotherapy, after the 3rd cycle, and at 2 weeks and 3 months after chemotherapy. Cumulative changes of serum AMH levels in patients who received different chemotherapy regimens were analyzed.

**Conclusions:**

Chemotherapy for GTN affects the ovarian reserve, with substantial differences between chemotherapy protocols. The results improve our understanding of ovarian toxicity and support the use of fertility preservation strategies.

## INTRODUCTION

Gestational trophoblastic disease (GTD) comprises a group of disorders in which tumors arise from placental trophoblastic tissue after abnormal fertilization. The malignant forms of GTD are collectively known as gestational trophoblastic neoplasias (GTNs) and include invasive mole, choriocarcinoma, placental site trophoblastic tumor (PSTT), and the extremely rare epithelioid trophoblastic tumor [1]. GTNs are highly responsive to chemotherapy even with distant metastasis. Improvements in management and follow-up protocols have increased the overall cure rate to over 98% with fertility retention, whereas most women would have died from malignant disease 60 years prior [2]. While curing the disease, chemotherapy agents cause depletion of the primordial follicle pool, affecting ovarian function. This has been demonstrated in breast cancer [3], lymphoma [4], and ovarian germ cell tumors [5].

Anti-Müllerian hormone (AMH) is a member of the transforming growth factor β (TGF-β) family of growth and differentiation factors [6]. This hormone is released by the granulosa cells of antral and pre-antral follicles and is predominantly known for its role in embryogenesis. Serum AMH levels have been shown to be proportional to the number of developing follicles [7]. AMH can be used to monitor the effect of chemotherapy on the ovarian reserve, a woman’s reproductive potential reflected by the number and quality of remaining oocytes [8]. According to recent studies, AMH levels decline to different extents in female cancer survivors [3–5, 9]. In the present study, we monitored changes in serum AMH concentrations during chemotherapy and a 3-month follow-up to determine gonadal toxicity of different chemotherapy regimens and assess short-term AMH level recovery.

## RESULTS

### Study population

Table 1 shows the patient characteristics. A total of 34 women (mean age, 30.8 ± 6.2 years; range, 23–45 years) with a diagnosis of invasive mole (*n* = 27) or choriocarcinoma (*n* = 7) were recruited before initiation of chemotherapy. In 20 of the 27 patients with invasive mole, treatment began with dactinomycin D (Act-D) and was changed in 7 cases to FAV (floxuridine, dactinomycin, vincristine), FAEV (floxuridine, dactinomycin, etoposide, vincristine), or EMA/CO (etoposide, methotrexate, dactinomycin, cyclophosphamide [CTX], vincristine) because of resistance to Act-D. The remaining 7 patients with invasive mole received combination chemotherapy (FAV in 6 cases and FAEV in 1). The 7 patients with choriocarcinoma were treated with a regimen that included etoposide (FAEV or EMA/CO).

**Table 1:** Demographic, histological and treatment characteristics

Characteristics	NO. (Ratio) or Mean (standard deviation) or Median (range)
**Demographic characteristics**	
Age at diagnosis (y)	30.8 ± 6.2
< 30 years	19 (55.9%)
30–35 years	10 (29.4%)
> 35 years	5 (14.7%)
BMI (kg/m^2^)	22.9 ± 3.2
Gravidity history	2 (1–6)
Parity history	1 (0–1)
FIGO score	
≤ 6	22 (64.7%)
≥ 7	12 (35.3%)
**Treatment characteristics**	
Chemotherapy regimens	
Act-D only	13
Act-D changed to combination chemotherapy	7
combination chemotherapy	14
Number of chemotherapy cycles	6 (3–13)

### Basal serum AMH/FSH levels and distribution according to age

**Figure 1:**
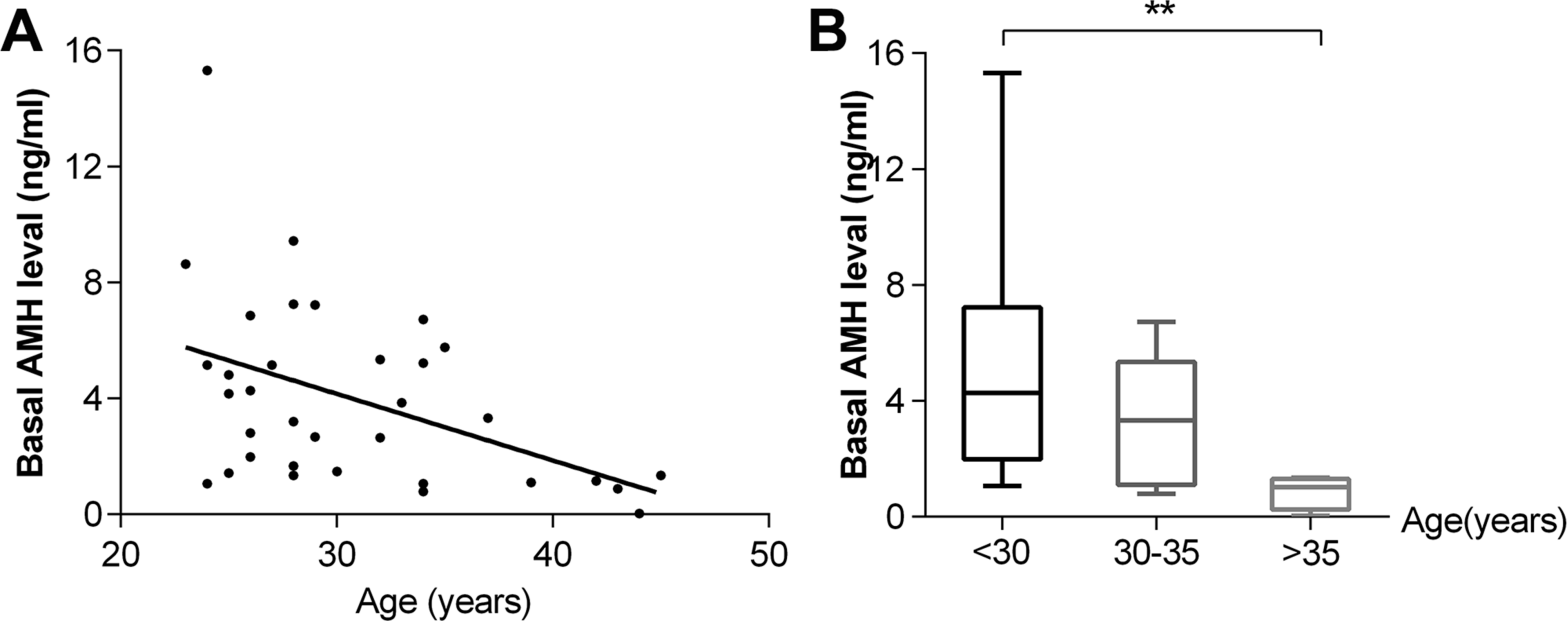
Correlation between basal AMH level and age (**A**) and distribution of AMH levels according to age groups (**B**) (*n* = 34). ^**^p < 0.001.

**Figure 2:**
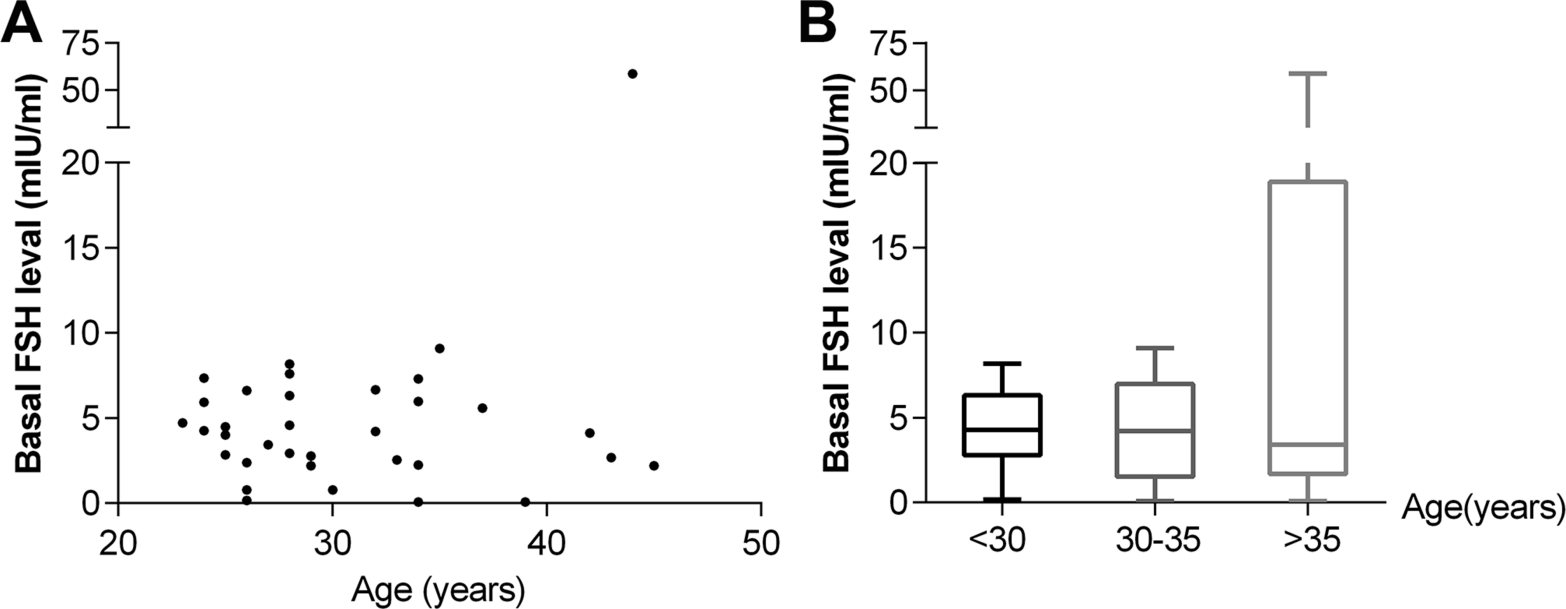
Correlation between basal FSH level and age (**A**) and distribution of FSH levels according to age groups (**B**).

At diagnosis, the mean basal AMH level was 3.98 ± 3.20 ng/mL (median, 3.27 ng/mL), and the mean basal FSH level was 5.71 ± 9.69 mIU/mL (median, 4.18 mIU/mL). A significant correlation was found between basal AMH level and patient age at diagnosis (r^2^ = 0.1996, *p* = 0.0081) (Figure 1A). The median AMH level was significantly lower in women > 35 years of age (*n* = 5) than in women < 30 years of age (*n* = 19) (*p* = 0.001) (Figure 1B). The basal FSH level did not correlate with age (*p* = 0.0575) (Figure 2A), and there was no significant difference in median FSH level among the three age groups(< 30 years, 30–35 years and >35 years) (*p* = 0.8499) (Figure 2B).

### Serum AMH/FSH levels after 3 chemotherapy cycles

**Figure 3:**
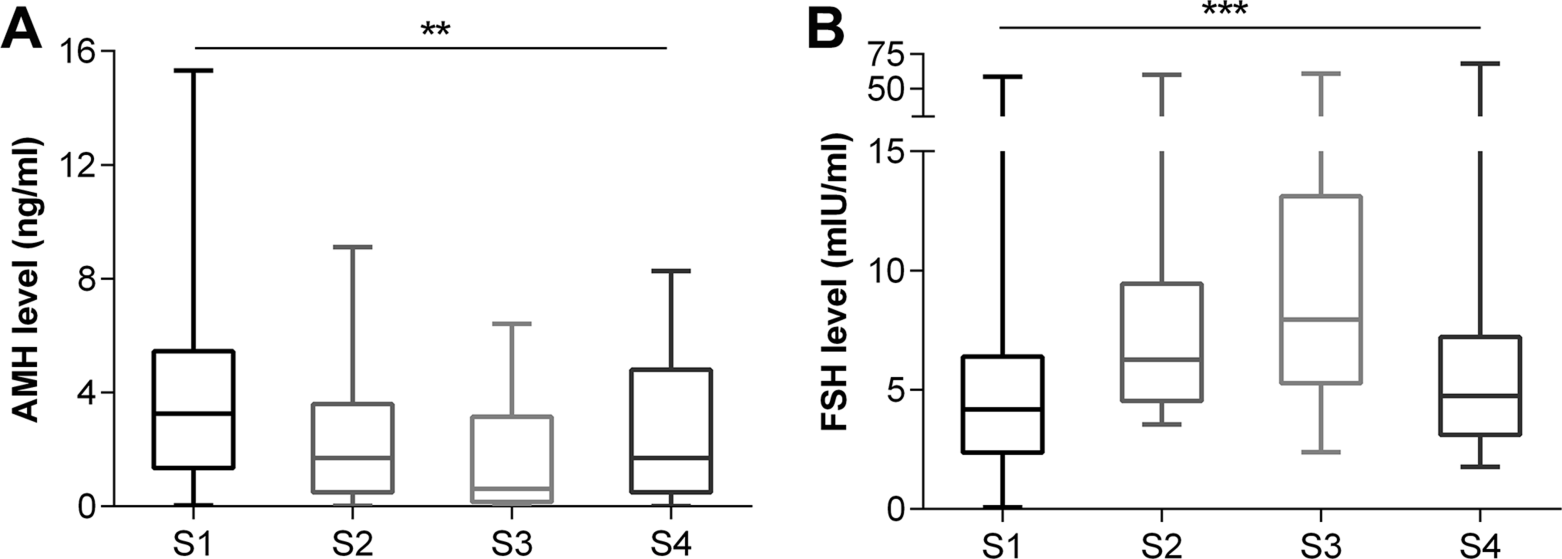
Evolution of serum AMH (**A**) / FSH (**B**) level at each visit. S1: at diagnosis, S2: after the 3rd chemotherapy cycle, and S3–4: 2 weeks and 3 months after completion of chemotherapy treatment. ^**^p < 0.001, ^***^p < 0.0001.

**Figure 4:**
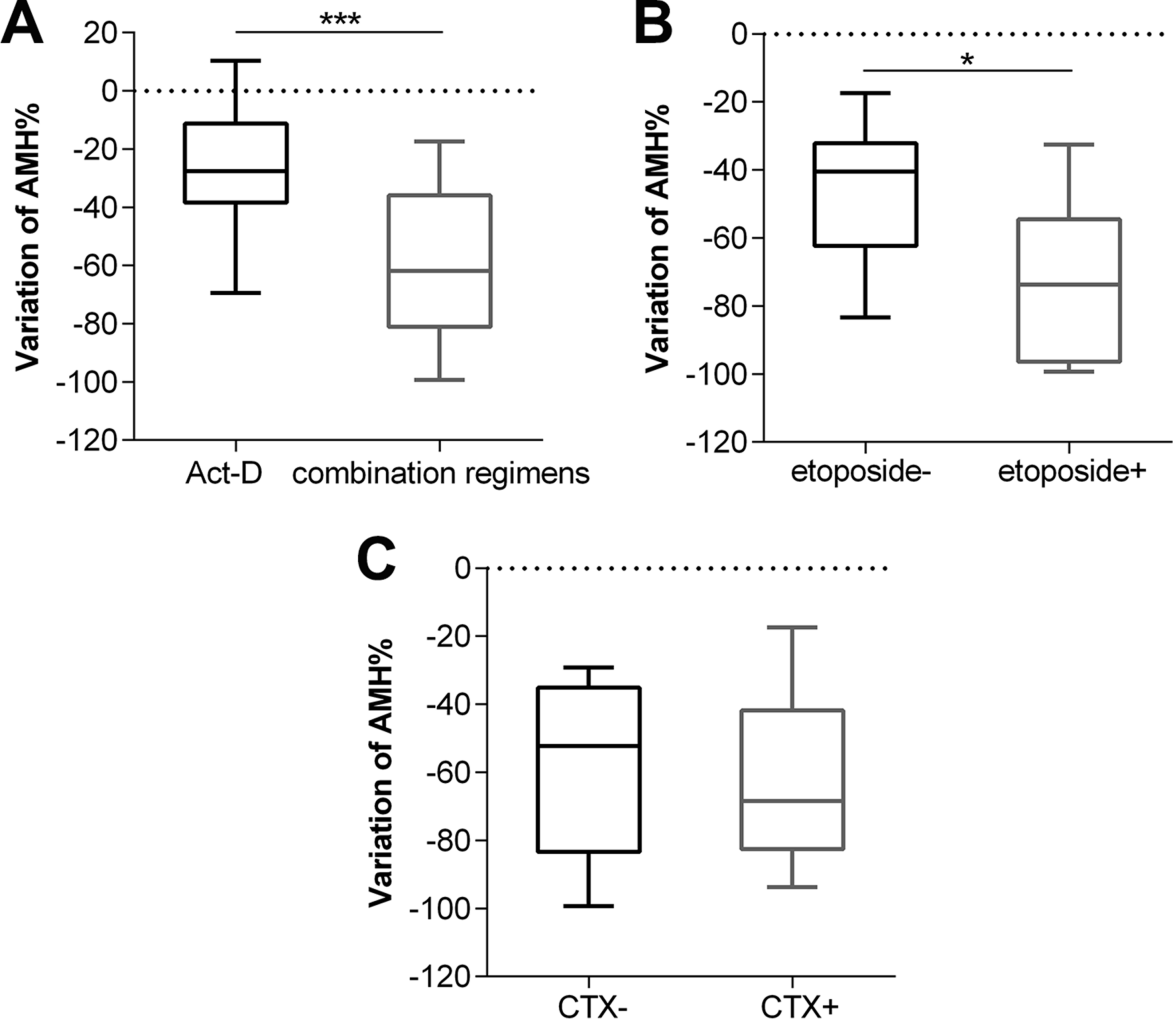
Cumulative variation of AMH between patients with combination chemotherapy and Act-D regimen (**A**), and patients used combination regimens with etoposide or not (**B**), and patients used combination regimens with CTX or not (**C**) after 3 cycles chemotherapy. CTX, cyclophosphamide. ^*^p < 0.01, ^***^p < 0.0001.

After 3 cycles, serum AMH levels decreased in 31 patients and slightly increased in only 3, all of which received Act-D. There was a significant difference between AMH levels after 3 cycles and the basal AMH levels (median: 3.27 vs. 1.70 ng/mL) (*p* < 0.0001) (Figure 3A). FSH levels increased significantly after 3 cycles (median: 4.18 vs. 6.26 mIU/mL) (*p* = 0.0002) (Figure 3B). The magnitude of the AMH level decline in the combination chemotherapy group (*n* = 17) was greater than in the Act-D-only group (*n* = 17) (27.57% vs. 61.80%) (*p* = 0.0004) (Figure 4A), while no such trend was observed for the FSH level (*p* = 0.2870). In the combination chemotherapy group, the magnitude of AMH level decline was 73.69% in patients treated with regimens including etoposide (FAEV or EMA/CO) (*n* = 8) and 40.51% in patients who did not receive etoposide (FAV) (*n* = 9) (*p* = 0.0359) (Figure 4B). The magnitude of AMH level decline in patients whose regimens included CTX (FAEV or EMA/CO) (*n* = 5) was not significantly different from that in patients who received regimens without CTX (FAV or FAEV) (52.28% vs. 68.41%, respectively, *p* = 0.6267).

### Chemotherapy completion and follow-up

After the completion of chemotherapy, all patients achieved complete remission. A total of 10 patients underwent hysterectomy and bilateral salpingectomy because of intramural lesions when the level of β hCG was normal or close to normal. Of these, 9 were treated with a combination regimen. The serum AMH levels showed a further decline at 2 weeks after chemotherapy completion, as compared to the levels after the 3rd cycle (*p* < 0.0001). The median AMH level after chemotherapy completion was 0.62 ng/mL (Figure 3A). In contrast, FSH levels increased (*p* = 0.0010) with a median value of 7.95 mIU/mL (Figure 3B). The average cumulative decrease in AMH level was 64.47% (95% CI: 54.92—74.02%) at the end of the chemotherapy cycles. The decrease was more obvious in the combination chemotherapy group (*n* = 21) than in the Act-D-only group (*n* = 13) (81.51% vs. 41.63%) (*p* < 0.0001). Multiple linear regression analysis to predict cumulative variation of AMH level yielded partial regression coefficients of 1.921 for the dose of vincristine (/mg) (*p* = 0.009) and 5.274 for the dose of etoposide (/g) (*p* = 0.032). No significant associations were found with age; BMI; use of fluorouracil, dactinomycin, methotrexate, or cyclophosphamide; and history of hysterectomy and bilateral salpingectomy.

**Figure 5:**
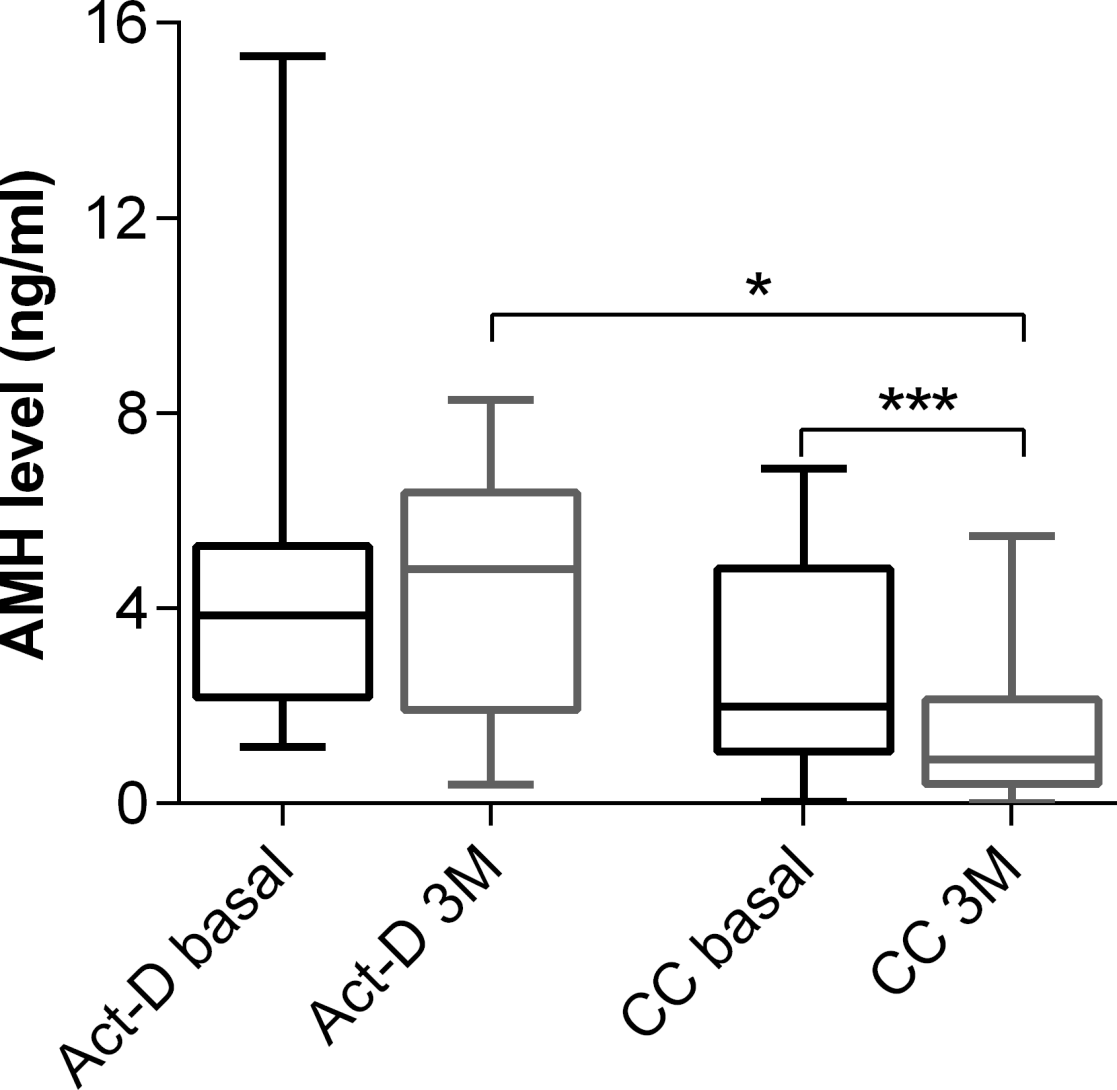
Serum AMH levels between Act-D group and combination chemotherapy group at diagnosis and 3-month follow-up CC, combination chemotherapy ^*^p < 0.01, ^***^p < 0.0001.

Three months after chemotherapy completion, 24 (70.6%) patients attended a follow-up appointment at our hospital and provided a blood sample. Serum AMH levels recovered partially or completely in all patients except one. The increase in AMH level was much greater in the Act-D group (*n* = 9) than in the combination chemotherapy group (*n* = 15) (*p* = 0.0067). The serum AMH levels in 3 months follow-up were not significantly different from the basal levels in the Act-D group (*p* = 0.4961). In contrast, they were still significantly lower than the basal levels in the combination chemotherapy group (*p* < 0.0001) (Figure 5). While cycles of those two groups had no significant difference (*p* = 0.3296). Moreover, the serum AMH levels in the combination chemotherapy group were lower than those in the Act-D group at 3 months (*p* = 0.0122) (Figure 5). The final AMH level and the corresponding cumulative decrease at the end of the study (3 months after chemotherapy completion) were 2.56 ± 2.45 ng/mL and 32.50% (95% CI: 17.56–49.63%), respectively. In the recovery period, FSH levels decreased in 18 patients (75%), while in 6 patients (25%) those continued to increase. The FSH levels at the 3-month follow-up (4.74; 95% CI, 2.45–13.75) were significantly lower than at 2 weeks after chemotherapy completion (7.95; 95% CI, 8.41–19.04) (*p* = 0.029). Nevertheless, those were still higher than the basal levels (4.18; 95% CI, 2.33–9.09), although the difference was statistically insignificant (*p* = 0.22) (Figure 3B).

### Menstrual status

The prevalence of chemotherapy-related amenorrhea (CRA) was 20.6% (*n* = 7) during chemotherapy. Of these 7 patients, 5 received combination regimens EMA/CO and 2 received Act-D only. CRA was not associated with age or basal AMH level (*p* = 0.3949 and 0.8431, respectively). At the 3-month follow-up after chemotherapy, all patients who had not undergone hysterectomy and bilateral salpingectomy had a regular menstrual cycle. Of these, 8 had normal menstrual flow, 14 had decreased flow, and 2 had slightly increased flow.

## DISCUSSION

Chemotherapy for many cancers is gonadotoxic, often decreasing the ovarian reserve and resulting in infertility or amenorrhea. This has been shown in patients with hematological malignancy [10] and breast cancer [3], and the degree of ovarian toxicity is related to the type of chemotherapy [11], dosage [12], and patient age [13]. GTN is a rare but curable disease, which is highly sensitive to chemotherapy. Almost all lesions are low-risk, and 80% to 90% of high-risk lesions are eventually cured [14]. Given the improved prognosis of women with GTD, preservation of their fertility has gained importance in recent years. However, studies focusing on ovarian function in patients with GTN have been sparse.

In this observational prospective cohort study, we assessed changes in serum AMH and FSH levels from the time of diagnosis to 3 months after chemotherapy completion in 34 premenopausal women with GTN who received different chemotherapy regimens depending on FIGO staging. We found that basal AMH level but not FSH level at diagnosis had a significant correlation with patient age. Helden et al. [13] showed that the decrease of both median and mean AMH levels was highly correlated with advancing age in a group of presumably healthy women of reproductive age. Dezellus et al. [3] found a negative correlation between AMH level before chemotherapy and age. Our results are concordant with these previous findings. Thus, serum AMH levels decreased progressively in the course of chemotherapy. In this regard, Iwase et al. [11] reported that serum AMH levels were significantly lower in patients with GTN who underwent chemotherapy than in patients with hydatidiform mole who did not receive chemotherapy. Furthermore, Dezellus et al. [3] showed that serum AMH levels decreased after the first chemotherapy cycle and declined rapidly and steadily in all patients during chemotherapy.

The type of chemotherapy agent also influences the degree of decrease in ovarian reserve. Our patients at a low risk of metastatic GTN were treated initially with a single agent such as methotrexate or Act-D. According to a Cochrane Systematic Review that included seven randomized controlled trials, act-D is more likely to lead to primary cure than methotrexate in women with low-risk GTN [15]. We found a significant difference in cumulative decrease in serum AMH levels between patients receiving Act-D only and combination chemotherapy. Furthermore, the AMH level recovered faster in the Act-D-only group than in the combination chemotherapy group. In addition, Act-D single-agent chemotherapy had little adverse effects on ovarian reserve at the 3-month follow-up. This supports the use of Act-D in patients with low-risk GTN. Combination chemotherapy primarily included FAV, FAEV, and EMA/CO. Etoposide is considered a key agent for treatment of high-risk GTN, whereas EMA/CO is the most commonly used first-line combination chemotherapy regimen in such cases [16] and can be used in the management of refractory low-risk GTN [17]. 5-fluorouracil-based multidrug chemotherapy, which has been used at the Peking Union Medical College Hospital for several decades, can be an effective initial treatment for GTN [18]. FAEV is effective for high-risk, drug-resistant, and relapsed GTN [19] and is a primary treatment regimen for stage IV GTN [20]. A Japanese study [11] found that serum AMH levels at follow up were significantly lower in patients who had received etoposide-positive regimens than in those who had received etoposide-negative regimens. In the current study, the magnitude of decrease in AMH levels was larger in patients who received regimens including etoposide (FAEV and EMA/CO) than in patients who were not treated with etoposide (FAV). Moreover, in multiple linear regression analysis, cumulative variation in AMH level decline was associated with doses of etoposide and vincristine. CTX, an alkylating agent, is known to be highly gonadotoxic. Rosendahl et al. [12] reported significantly lower AMH levels in patients who received alkylating agents, whereas in the study of Dezellus et al. [3] 231 of 250 patients with breast cancer (92.4%) who received CTX-positive regimens had CRA at the end of chemotherapy. Although 5 of the 7 patients (71.4%) who received EMA/CO in the current study experienced CRA, we did not find any correlation between CTX dose and changes in AMH level.

FSH has been widely used as an ovary function marker for many years. In our study, the median FSH levels were not significantly different in three age groups of premenopausal women. After 3 cycles of chemotherapy, the FSH level increased significantly and was higher at the end of chemotherapy. However, the FSH level increase was not significantly different between the Act-D group and the combination chemotherapy group. Unlike AMH levels, serum FSH levels recovered to the basal values during the 3-month follow-up. It was shown before that FSH level can only be used to differentiate between patients with < 5 follicles and >19 follicles. Therefore it can only reflect a considerable loss of ovarian function [21]. Since the menstrual cycle of patients with GTN before chemotherapy is influenced by the β hCG level, the FSH level may be of limited use for assessing ovarian reserve during chemotherapy considering that it is usually measured during the early follicular phase.

During chemotherapy, amenorrhea occurred in 7 of 34 patients (20.6%), while 3 months after chemotherapy all patients who had not undergone hysterectomy had a regular menstrual cycle. A retrospective study that analyzed women treated with chemotherapy for GTN between 1986 and 2012 showed that resumption of menses after chemotherapy occurred in 100% of the 12 high-risk and 97% of the 33 low-risk patients [22]. These results are consistent with the findings of our study. Furthermore, in the mentioned study, 8 high-risk women (53%) and 29 low-risk women (85%) eventually became pregnant [22]. Lok et al. [23] reported that 12 of 14 patients with GTD (86%) treated with EMA/CO successfully conceived. Another study showed that 3 patients with GTN had very low AMH levels 4–13 months after multiagent chemotherapy but spontaneously conceived within 2–9 months [24]. Whether chemotherapy regimens and AMH levels correlated with fertility could not be determined in the current study because of the small sample size, which constitutes a limitation of the present work.

In conclusion, to the best of our knowledge, this is the first prospective study that used serum AMH levels to assess ovarian reserve during chemotherapy in patients with GTN. Both Act-D and combination chemotherapy affected ovarian reserve, but it recovered after 3 months in patients who received Act-D only and failed to recover in those who received combination chemotherapy. Regimens that included etoposide caused more damage to the ovarian reserve. Thus, measurement of the AMH level before treatment may help guide the selection of the chemotherapy regimen and the decision whether to consider fertility preservation. Future studies should evaluate the relationship between pre- and post-chemotherapy serum AMH levels with reproductive outcomes in patients with GTN.

## MATERIALS AND METHODS

### Patients

Patients who underwent chemotherapy for GTN at Peking Union Medical College Hospital from October 2015 to December 2016 were recruited for this observational prospective cohort study. The inclusion criteria were as follows: (1) age ≤ 45 years, (2) no history of chemotherapy, (3) no evidence of endocrine disorders, including polycystic ovarian syndrome, thyroid dysfunction, hyperprolactinemia, or Cushing’s syndrome. The exclusion criteria were current hormonal treatment, including oral contraceptives, and previous ovarian or pituitary surgery. This study was approved by the ethics committee of Peking Union Medical College Hospital. All patients provided written informed consent before the study.

### Diagnosis and chemotherapy regimens

The level of serum human chorionic gonadotropin β (β hCG) was measured for all patients. Computed tomography (CT) or magnetic resonance imaging and ultrasonography of the pelvis were performed. Chest radiography and CT were used to detect pulmonary metastasis. A detailed physical examination, including pelvic examination, was performed for each patient. All patients were scored according to the International Federation of Gynecology and Obstetrics (FIGO) staging system and classified into low-risk and high-risk GTN groups [25]. The chemotherapy regimens were designed based on the FIGO score. Act-D was administered to patients with a low-risk invasive mole. Combination chemotherapy regimens were used to treat patients with high-risk invasive GTN and choriocarcinoma and patients with low-risk invasive GTN who showed resistance to Act-D. Combination chemotherapy regimens included FAV, FAEV, and EMA/CO.

### Hormone level measurements

A medical interview and blood sampling for determining AMH and follicle stimulating hormone (FSH) levels were performed for each patient at diagnosis (sample S1), after the 3rd chemotherapy cycle (sample S2), and 2 weeks and 3 months after completion of the chemotherapy (samples S3 and S4, respectively). Samples S1–S3 were obtained on a random day of the menstrual cycle, whereas samples S4 were obtained on day 2–7 of the menstrual cycle if the patient had regular menstrual bleeding. For each sample, serum was separated from the whole blood by centrifuging at 3000 rpm for 10 minutes, transferred to a sterile polypropylene tube, and stored at –80°C until assayed. AMH and FSH levels were measured using an automatic chemiluminescence immunoassay analyzer (Beckman Coulter UniCel DXI800, Brea, CA, USA) and the corresponding reagents, calibration materials, and quality control materials. For the AMH assay (Access AMH, cat. no. B13127; Beckman Coulter), the intra-assay and inter-assay coefficients of variation were below or equal to 1.7% and 2.8%, respectively. For the FSH assay (Access hFSH, cat. no. 33520; Beckman Coulter), the intra-assay and total coefficients of variation were below or equal to 4.3% and 5.6%, respectively.

### Statistical analysis

Significant differences between groups were detected using the Kruskal-Wallis and Mann–Whitney *U* tests. The Friedman and Wilcoxon tests were used to detect significant variations in the tested parameters at different time points. The Spearman coefficient was used for correlation studies. Cumulative variation was predicted using multiple linear regression. The Statistical Package for Social Sciences, version 11.5 (SPSS Inc., Chicago, IL, USA), and GraphPad Prism 6 (GraphPad Software Inc., USA) were used for statistical analysis. A *p* value < 0.05 was considered to represent a statistically significant difference.

## Abbreviations

AMH: anti-Müllerian hormone
CRA: chemotherapy-related amenorrhea
CT: computed tomography
Act-D: dactinomycin D
EMA/CO: (etoposide, methotrexate, dactinomycin, cyclophosphamide, vincristine)
FAEV: (floxuridine, dactinomycin, etoposide, vincristine)
FAV: (5-FU, dactinomycin, vincristine)
FIGO: International Federation of Gynecology and Obstetrics
FSH: follicle-stimulating hormone
β hCG: human chorionic gonadotropin β
GTD: gestational trophoblastic disease
GTN: gestational trophoblastic neoplasia
PSTT: placental site trophoblastic tumor
TGF-β: transforming growth factor β
